# Supplementary figures and images for: Cell death induced in glioblastoma cells by Plasma-Activated-Liquids (PAL) is primarily mediated by membrane lipid peroxidation and not ROS influx
Source: PLoS One. 2022 Sep 22;17(9):e0274524. doi: 10.1371/journal.pone.0274524 (PMC9498962; doi:10.1371/journal.pone.0274524)

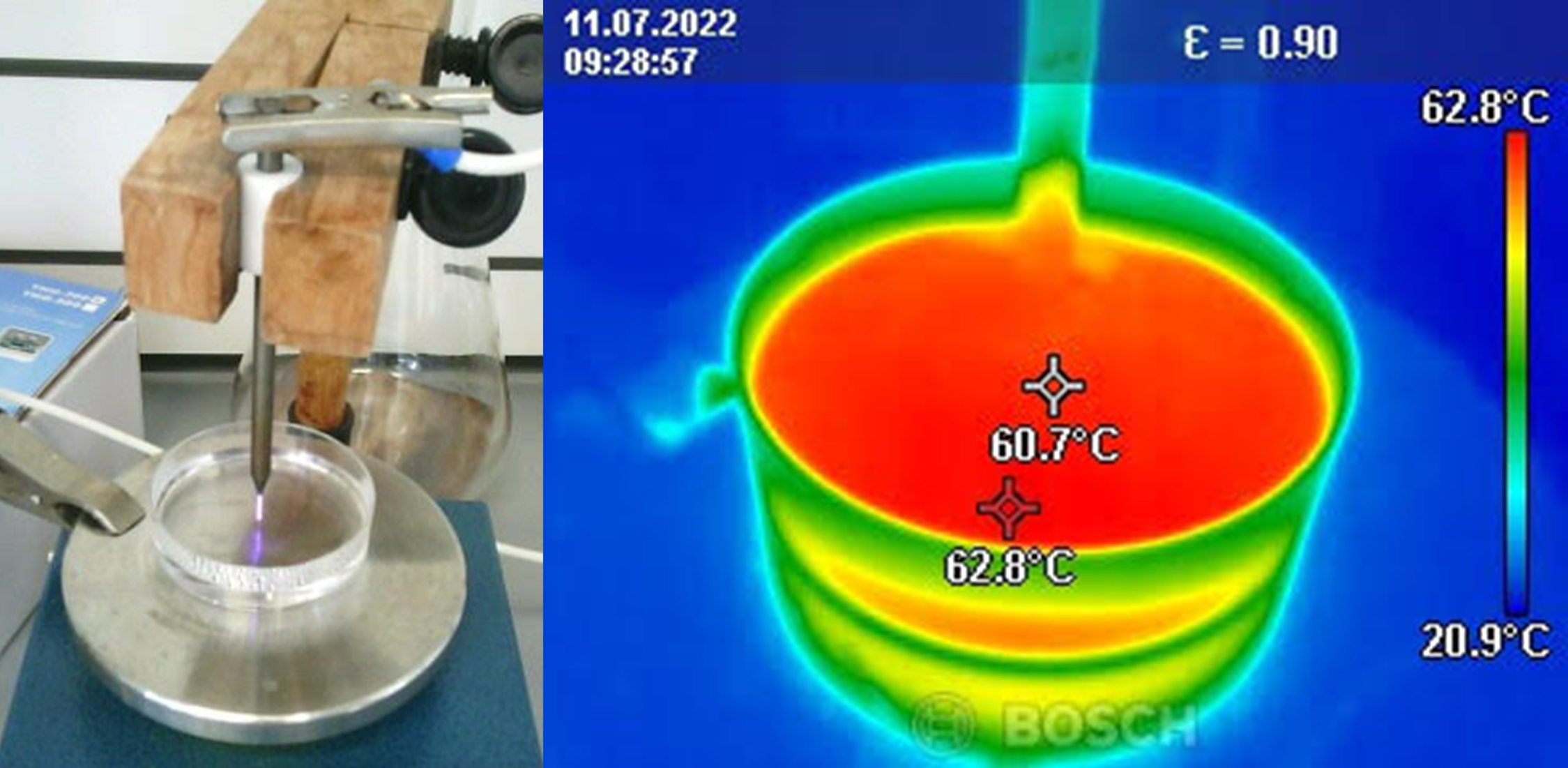

Supplement: S1 Fig — In this study, 10 min of plasma treatment was applied to 10 mL of water. The thermal measurement was taken at the end of the treatment. (TIF) [file pone.0274524.s001.tif]

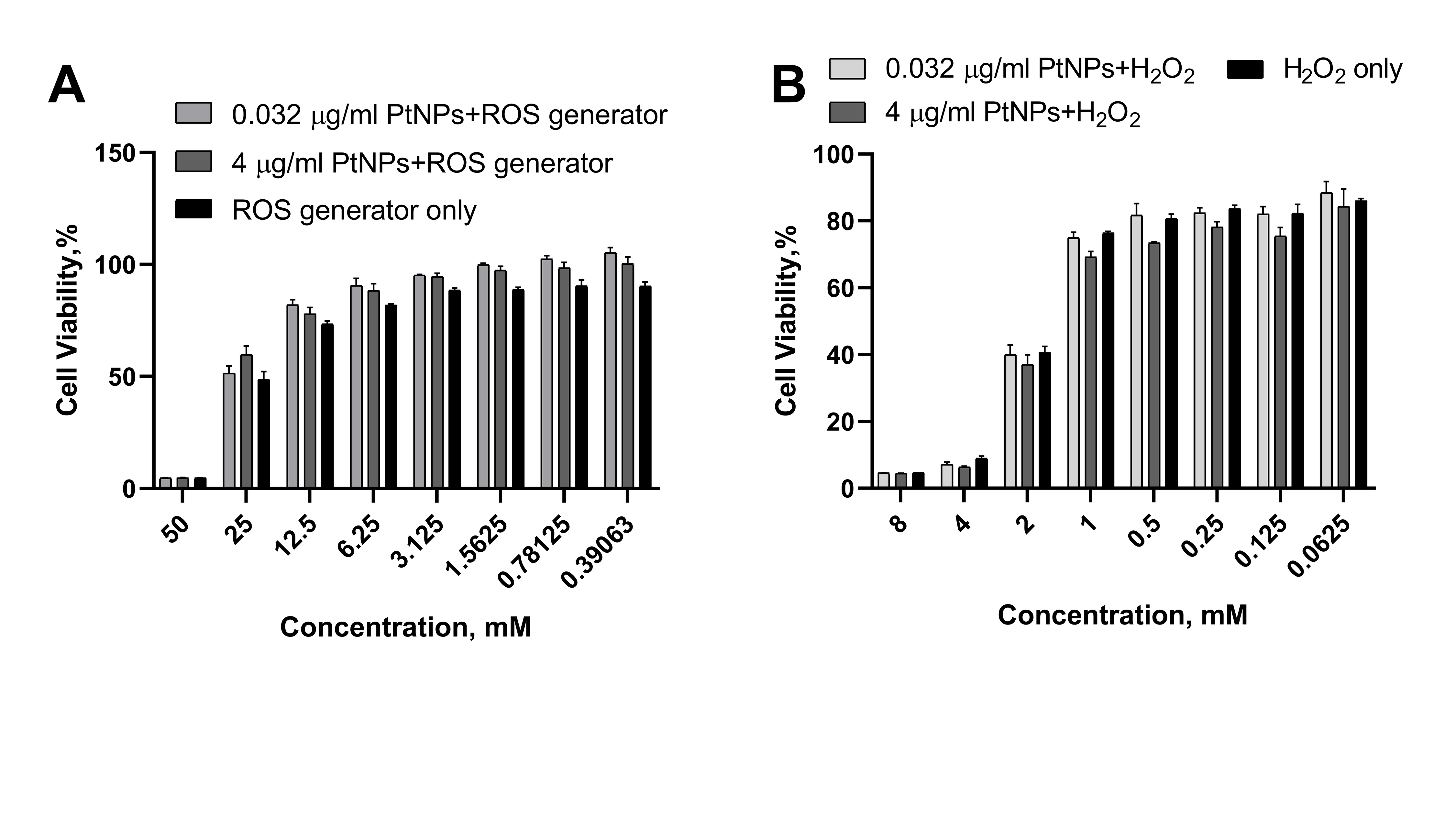

Supplement: S2 Fig — (A) U-251 MG cells were incubated with 0.032 and 4 μg/ml PtNPs for 4 h and then treated with increasing concentrations (0 ≤ 50 mM) of ROS generator. (B) U-251 MG cells were incubated with 0.032 and 4 μg/ml PtNPs for 4 h and then treated with increasing concentrations (0 ≤ 8 mM) of H2O2. Alamar blue cell viability assay was carried out 4 h after treatment. (TIF) [file pone.0274524.s002.tif]

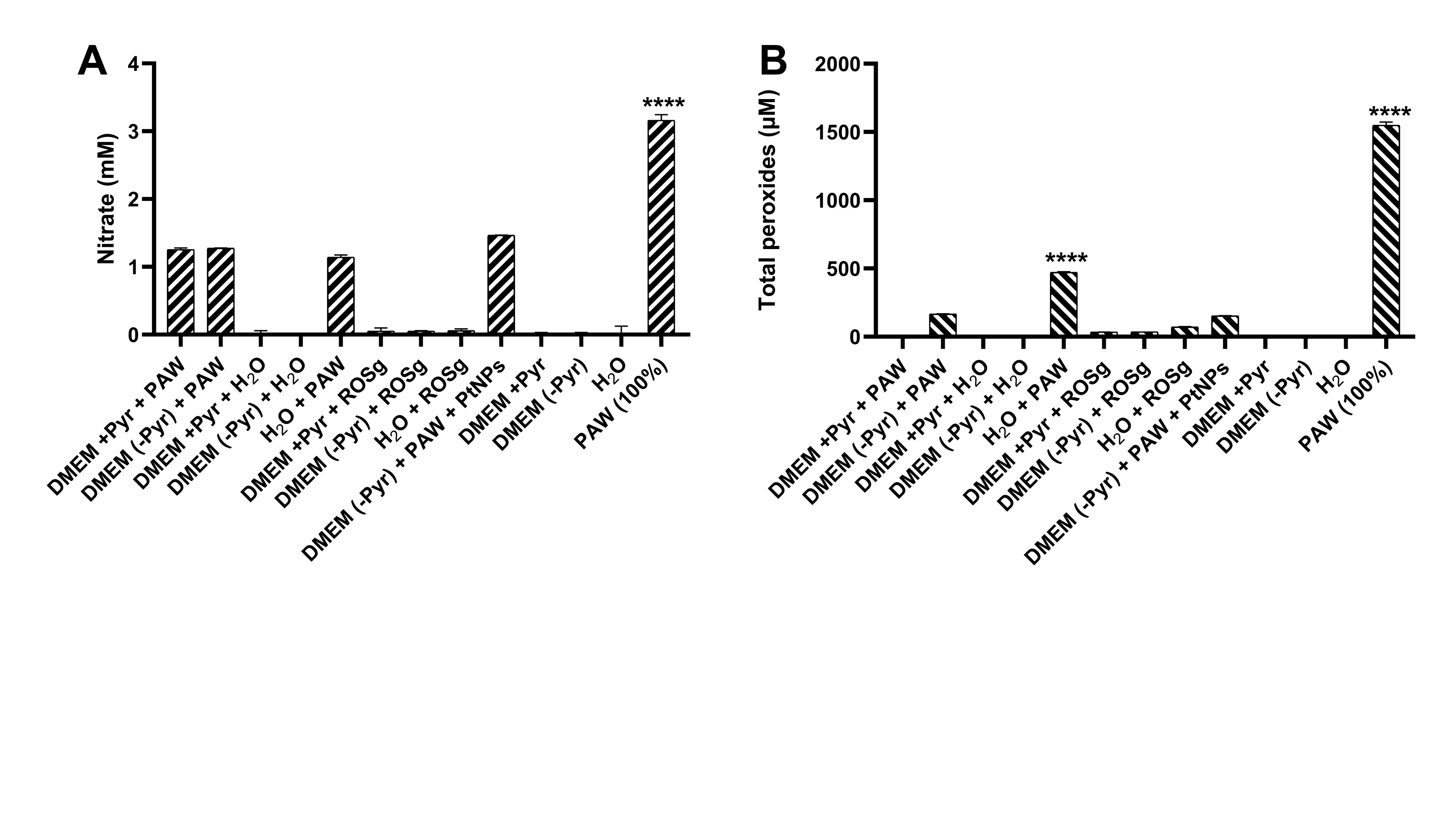

Supplement: S3 Fig — DMEM-DMEM with 10% FBS used for cell culture; Pyr -Pyruvate; ROSg—ROS generator; In this experiment, 30% of PAW was selected as the representative condition. The PAW was diluted in DMEM with or without pyruvate, DMEM without pyruvate but with 4 μg/ml PtNPs and water. 25 mM of ROS generator in DMEM with/without pyruvate and H2O was also tested as controls. (TIF) [file pone.0274524.s003.tif]

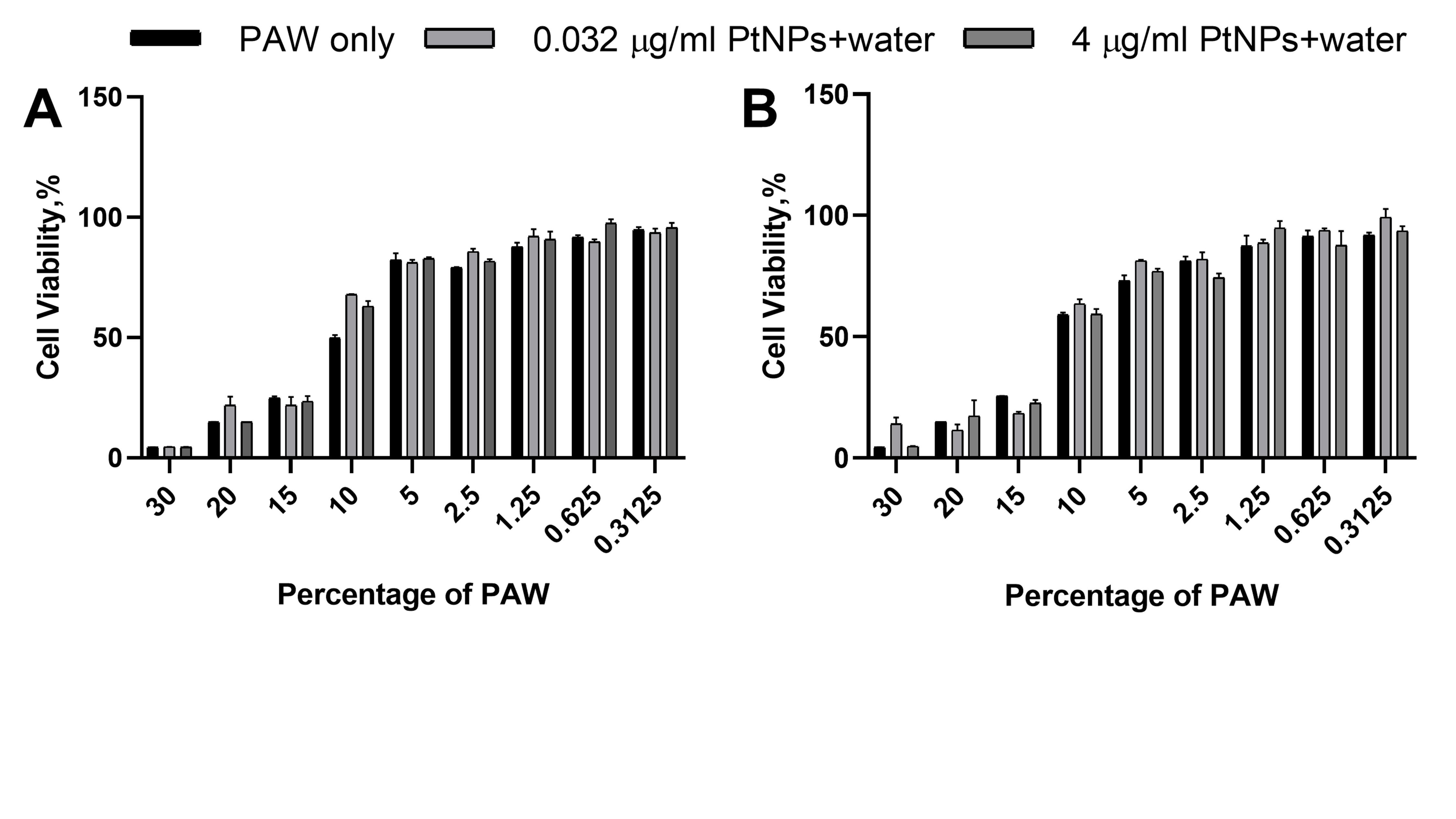

Supplement: S4 Fig — U-251 MG cells were treated with 0.032 and 4 μg/ml PtNPs and increasing concentrations (0 ≤ 30%) of PAW simultaneously and incubated for 24h (A) or 48 h (B). (TIF) [file pone.0274524.s004.tif]
