# Supplementary material for: Cell death induced in glioblastoma cells by Plasma-Activated-Liquids (PAL) is primarily mediated by membrane lipid peroxidation and not ROS influx
Source: PLoS One. 2022 Sep 22;17(9):e0274524. doi: 10.1371/journal.pone.0274524 (PMC9498962; doi:10.1371/journal.pone.0274524)
Supplement: S1 Table — (DOCX) [file pone.0274524.s005.docx]

**S1 Table. ROS and RNS characterisation of PAW generated after 10 min treatment.**

| Species | Measured concentration | Measurement Methods |
| --- | --- | --- |
| Total oxidising species | Around 1.18 mM | KI method |
| Hydrogen Peroxide | Around 0.88 mM | TiOSO4 method |
| Nitrate | Around 3.73 mM | DMP method |
| Nitrite | Not detectable | Griess method |
