# Supplementary material for: Cell death induced in glioblastoma cells by Plasma-Activated-Liquids (PAL) is primarily mediated by membrane lipid peroxidation and not ROS influx
Source: PLoS One. 2022 Sep 22;17(9):e0274524. doi: 10.1371/journal.pone.0274524 (PMC9498962; doi:10.1371/journal.pone.0274524)
Supplement: Attachment — Submitted filename: Response to Reviewers b.docx [file pone.0274524.s006.docx]

**REF: PONE-D-22-14482 Cell death induced in Glioblastoma cells by Plasma-Activated-Liquids (PAL) is primarily mediated by membrane lipid peroxidation and not ROS influx**

Dear Editor and Reviewers,

Thank you very much for your comments and suggestions. We have found these to be very helpful to further improve our manuscript. We have addressed every comment below, and have updated the manuscript accordingly. Please find the point by point response (colour coded) below.

With best wishes,

James, Sebnem and co-authors

**Review Comments to the Author**

**Reviewer #1:** In this manuscript, the authors try to identify potential antagonistic effect between antioxidative intracellularly accumulated platinum nanoparticles (PtNPs) and PAL. It is found that PAL can significantly reduce the viability of glioblastoma U-251MG cells and it did not involve measurable ROS influx but instead lead to lipid damage on the plasma membrane of cells exposed to PAL. In addition, it is found that the intracellular antioxidative PtNPs showed no protective effect against PAL. This study contributes to further understanding of principle cell killing routes of PAL and discovery of potential PAL-related therapy and methods to inhibit side effects. This obtained results are very interesting. The paper is well organised and nice written. Thus I would recommend publish it after the authors address several of my minor comments:

1)Since this paper is about using plasma jet for cancer applications, there are several nice review paper on plasma jet and cancer therapy such as (1) Phys. Rep. 630, 1-84 (2016), (2) Int. J. Cancer 134, 1517 (2014), and (3) Scientific Reports, 7, 4479, (2017).(4) Phys. Plasmas 28, 100501 (2021) should be cited and discussed.

Many thanks to the Reviewer for the suggestion. We have included the discussion based on the recommended papers in the Discussion section 1^st^ and 2^nd^ paragraphs (Page 17-18, Line 360-381)

2)What's the diameter of the tip of the electrode?

It's around 1 mm, and we have included this information in the Method section (Page 6, Line 108-109)

3) What's temperature of the liquid after plasma treatment?

The liquid temperature is around 60 ℃ after 10 min plasma treatment. Then the PAW was cooled down to room temperature before mixing with culture media. We have included this detail in the Method section (Page 6, Line 110-111) and SI (S1 Figure)

4)Why choose the deionised water rather than culture medium?

Thank you for noting this point. We consider deionised water is a much simpler system than a culture medium, thus will be helpful to study the mechanism. In the future study, we will investigate in depth using plasma-activated water, saline, culture medium and other solutions for their application and eventually contribute to clinical plasma medicine.

5) What's the NO2-, NO3-, H2O2 and pH of the PAW?

There are around 3.7 mM of NO_3_- and 0.9 mM of H_2_O_2_ in the PAW following 10 min treatment with 10 ml water, however, no nitrite was detected using Griess method. We have measured and included the data in the Result section (Page 11, Line 222-228) and SI (S1 Table), while the quantification methods have been described in the Method Section (Page 6-7, Line 116-146). For the pH, we have reported in one of our previous articles, which is Applied Sciences, 2021, Distinct chemistries define the diverse biological effects of plasma activated water generated with spark plasma discharges, and we also have cited the data in the Result section (Page 11, Line 226-230).

6)Some of the figures are not clear, please improve it.

Thanks to the Reviewer, we have improved and rearranged all figures.

**Reviewer #2:** The paper deals with a study that is aimed to demonstrate if the cell-death mediated by Plasma Activated Water is due to ROS influx or membrane lipid peroxidation. For this purpose, the authors studied the use of PtNPs as potential intracellular ROS scavengers that showed their efficacy in previous experiments performed with direct plasma treatment instead of PAW application. The paper demonstrates that the presence of intra-PtNPs is not efficacious against ROS when cells are exposed to PTW. The paper in the opinion of the referee shows several experiments well described both in term of their rationale and results obtained but there are in the opinion of the reviewer some issues that need to be included in the paper that allow the author to better address their discussion and conclusion. Here below are reported a list of comments, suggestions:

1) The authors used the PAW whose chemical characterisation is included in table S1, but diluted in cell culture medium w/o pyruvate. Did the author quantify the concentration of H2O2, NO2- and NO3- after dilution in the cell culture? As well known the cell culture medium could actively contribute to scavange ROS as well. About that, the reviewer suggests to cite "Antioxidants 2021, 10(4), 605; <https://doi.org/10.3390/antiox10040605>" and similar papers showing the scavenging effect of cell culture media w/o FBS. Thus, in order to be sure that the observed results are connected to a successful scavenging of ROS by antioxidants used or that the use of ROS generator is efficacious in promoting the formation of ROS the reviewer suggests to include quantification of ROS before and after each step (i.e. before and after dilution, before and after addition of ROS generator, before and after addition of pyruvate etc. …)

Many thanks to the Reviewer for raising those points. Indeed, besides pyruvate, the common antioxidant used in the medium, several components in the culture medium may act as ROS scavengers. We have cited relevant papers and discussed this point in the Discussion Section (Page 19, Line 413-418). Also, as suggested, we have quantified H_2_O_2_, NO_2_^-^ and NO_3_^-^ concentrations before and after each critical step in the Discussion section (Page 19-20 and Line 418-427) and SI (S3 Figure).

2) In the opinion of the reviewer before addressing some conclusion about intracellular scavenging of ROS by PtNPs it is necessary to demonstrate the presence of such particles inside cells. Did the authors observe PtNPs inclusion?

Based on our current resources and available microscopy techniques and the size of the PtNPs used (3 nm), we are sorry that we cannot directly observe the accumulation of PtNPs in cells. Several articles have previously demonstrated that the PtNPs accumulate into cells using electron microscopes which we have cited and discussed in the Discussion section (Page 18, Line 382-395). Moreover, we have followed standard washing steps after PtNPs pre-incubation to ensure the remaining PtNPs are inside cells and residual extracellular PtNP are removed.

3) In the experiments in which the PtNPs are incubated together PAW are the authors sure that the nanoparticles are not affected in their structure by ROS. This aspect could, in the opinion of the referee compromise their efficacy in ROS scavenging. Also in this case it is necessary to quantify the concentration of ROS.

Thanks to the Reviewer for the suggestion. Indeed, the affections of PtNPs structures by ROS will be our follow-up work to investigate further. In this study, we have quantified the ROS concentration following the Reviewer's suggestion. The new data has been described in Discussion Section (Page 19-20, Line 418-427) and SI (S3 Figure), which is also mentioned in the responses to the comment 1.

4) Figure 4A and 4B: how the authors explain the data collected at PAW10%? These data in fact have a different trend respect the others because it seems that the addition of PtNPs decreases instead of increasing the cell viability.

We thank the Reviewer for noting this point. As seen from the reported data, we observed no sufficiently significant protection	provided by PtNPs against PAW in general. For Figures 4A and 4B, we repeated the experiments before submission but only presented those selected panels.

As the trend at PAW 10% is not significant in Figures 4A and 4B, we missed clarifying it. Furthermore, the repeated data showed that the addition of PtNPs barely changed the cell viability at PAW 10%, so now we have included the clarification and repeating data in SI. Please find the revision in the Discussion section (Page 20, Line 432-436) and SI (S4 Figure).

5) The statistical analysis of all data collected except for that one shown in figure 2 is not included. The reviewer suggests to insert statistical analysis of all reported data.

Many thanks to the Reviewer. We have included statistical analysis in all reported figures and/or figures captions.

6) Can the authors specify why used the concentration of H2O2 reported for the experiments reported in figure 8?

The incubation with 2 mM or similar concentrations of H_2_O_2_ has been used in peer-reviewed articles (https://doi.org/10.3390/ijms21197263; https://doi.org/10.1002/jbm.b.30360) and in our lab as positive controls that significantly lead to oxidative stress of cancer cell lines without immediate cell killing effects （~50% cell killing effect, S2 Fig）. Therefore, we chose this concentration in this study to highlight the influx of ROS and potentially different cell-killing mechanisms. We have now included the clarification following the Reviewer's suggestion in the Discussion section (Page 24, Line 520-531).

**Journal Requirements:**

When submitting your revision, we need you to address these additional requirements.

1. Please ensure that your manuscript meets PLOS ONE's style requirements, including those for file naming. The PLOS ONE style templates can be found at 
https://journals.plos.org/plosone/s/file?id=wjVg/PLOSOne_formatting_sample_main_body.pdf and 
https://journals.plos.org/plosone/s/file?id=ba62/PLOSOne_formatting_sample_title_authors_affiliations.pdf.

The manuscript has been updated to meet PLoS One style requirements.

2. Thank you for stating the following in the Acknowledgments Section of your manuscript: 
[This work is supported by TU DUBLIN Fiosraigh Research Scholarship programme (S.G.), Irish Research Council Government of Ireland Postdoctoral Fellowship Award GOIPD/2020/788 (Z.H., J.C.), Science Foundation Ireland Grant Numbers 14/IA/2626 (P.B, P.C., J.C.) 15/SIRG/3466 (DB), 16/BBSRC/3391 (PB) and 20/US/3678 (PB).]

We note that you have provided funding information that is not currently declared in your Funding Statement. However, funding information should not appear in the Acknowledgments section or other areas of your manuscript. We will only publish funding information present in the Funding Statement section of the online submission form. 
Please remove any funding-related text from the manuscript and let us know how you would like to update your Funding Statement. Currently, your Funding Statement reads as follows: 
 [This work is supported by TU DUBLIN Fiosraigh Research Scholarship programme (S.G.), Irish Research Council Government of Ireland Postdoctoral Fellowship Award GOIPD/2020/788 (Z.H., J.C.), Science Foundation Ireland Grant Numbers 14/IA/2626 (P.B, P.C., J.C.) 15/SIRG/3466 (DB), 16/BBSRC/3391 (PB) and 20/US/3678 (PB).]

Please include your amended statements within your cover letter; we will change the online submission form on your behalf.

We have removed funding information from the acknowledgements section and request that the Financial Disclosure section is changed to include the funding details below.

**The authors wish to acknowledge funding from:**

**Science Foundation Ireland (SFI)**

Grant Number 14/IA/2626 (PB,PC,JC)

Grant Number 15/SIRG/3466 (DB)

Grant Number 16/BBSRC/3391 (PB)

Grant Number 20/US/3678 (PB)

Grant Number 21/FFP-A/9189 (JC)

[www.sfi.ie](http://www.sfi.ie)

**Irish Research Council (IRC)**

Postdoctoral Fellowship Award GOIPD/2020/788 (ZH)

Enterprise Partnership Scheme EPSPG/2020/277 (BL)

<https://research.ie>

**TU Dublin**

Fiosraigh Research Scholarship Programme 2018 (SG)

[www.tudublin.ie](http://www.tudublin.ie)

3. We note that you have stated that you will provide repository information for your data at acceptance. Should your manuscript be accepted for publication, we will hold it until you provide the relevant accession numbers or DOIs necessary to access your data. If you wish to make changes to your Data Availability statement, please describe these changes in your cover letter and we will update your Data Availability statement to reflect the information you provide.

We have changed the data availability statement in the manuscript. We have uploaded all datasets to OSF and we will provide relevant DOI link prior to publication.
